# Simultaneous Noncentered Photoactivated Chromophore for Keratitis-Corneal Collagen Cross-Linking and Penetrating Keratoplasty for Treatment of Severe Marginal* Fusarium *spp. Keratitis: A Description of a New Surgical Technique

**DOI:** 10.1155/2017/6987896

**Published:** 2017-12-14

**Authors:** Kepa Balparda, Juan Carlos Mejia-Turizo, Tatiana Herrera-Chalarca

**Affiliations:** ^1^Department of Cornea and Refractive Surgery, Clínica de Oftalmología Sandiego, Medellín, Colombia; ^2^Department of Ophthalmology, Clínica de Oftalmología Sandiego, Medellín, Colombia; ^3^Universidad de Antioquia, Medellín, Colombia

## Abstract

The purpose of this article is to describe the use of simultaneous noncentered photoactivated chromophore for keratitis-corneal collagen cross-linking (PACK-CXL) combined with penetrating keratoplasty in the treatment of a severe marginal* Fusarium *spp. keratitis case with imminent corneal perforation. It is a retrospective case report study; it was performed by collecting clinical data, images, video, and postoperative evaluations. The clinical control of the infection was accomplished, despite difficulties in obtaining antifungal medications due to the patient's extremely poor socioeconomic status and essentially nonexistent health insurance. We can conclude that combining simultaneous decentered PACK-CXL with centered penetrating keratoplasty appears to be a safe and effective way of treating patients with fungal marginal keratitis with corneal perforation, in which regular penetrating keratoplasty alone would leave fungal elements in the receptor corneal tissue, which would predispose to infection of the graft.

## 1. Introduction

Fungal keratitis is a major concern for cornea surgeons, especially those in developing nations. Although topical antifungal therapy continues to be the gold-standard for treatment worldwide, photoactivated chromophore for keratitis-corneal collagen cross-linking (PACK-CXL) has been explored as an option for decreasing the likelihood of complications, specially corneal perforation.

When a corneal perforation does occur, an emergent tectonic penetrating keratoplasty (PK) is indicated. Nevertheless, a marginal keratitis with limbal involvement and a perforated central defect can represent a real challenge for the corneal surgeon. A regular PK leaves a small amount of infected receptor cornea in place, in which infectious agents can survive and infect the corneal graft, despite intensive topical antifungal treatment. A penetrating sclerokeratoplasty is not an easy option either, as it is a difficult-to-perform surgery, with a high rate of complications, including chronic glaucoma.

Studies in humans have demonstrated the ability of cross-linking to control corneal infections and stop melting in those patients in whom pharmacological management is ineffective. It could also decrease the probability of corneal perforation and the need for an emergent penetrating keratoplasty. Nevertheless, so far no paper has described PACK-CXL surgery immediately after PK once corneal perforation has ensued. In this paper, the authors present, to the best of their knowledge, the first published instance in which simultaneous PK and PACK-CXL were performed as treatment for a central corneal perforation with a marginal corneal compromise. This case highlights the possibility of PACK-CXL to help in the management of patients undergoing emergency PK in a context in which not all infectious burden can be removed. It also serves as a support to suggest that CXL could be safely undertaken immediately following PK, an approach which may be used for many other applications.

## 2. Case Report

**Figure 1:**
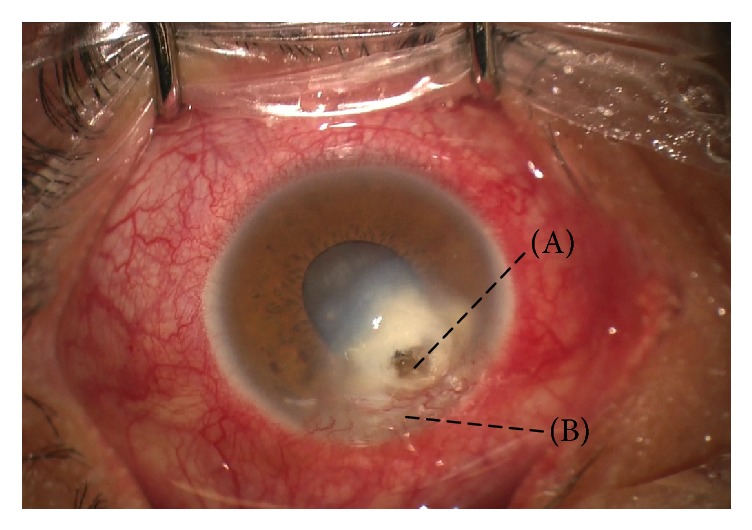
Clinical appearance of the patient before surgery. Please note a corneal perforation surrounded by a dense infiltrate (A) associated with inferior neovascularization (B).

A 39-year-old male patient, with no relevant personal history, presented to our cornea clinic complaining of a week duration of severe pain, tearing, redness, and blurred vision in the right eye. He worked as a farmer and janitor in a rural potato plantation in Colombia and had a history of working with soil and plants. On physical examination, his vision on the right eye was 20/400 (0.05, LogMAR 1.3). His right cornea had a 2 × 2 mm inferotemporal ulcer surrounded by a dense infiltrate that compromised all the way but 1.5 mm before the corneal limbus. Corneal thinning at the site of ulcer was about 50%. Clinical examination of his left eye was completely unremarkable. The patient was instructed to start hourly gatifloxacin 0.5% and hourly natamycin 5%. Sodium hyaluronate 4 mg/mL was also ordered. The patient presents to our clinic a week after medications were ordered. The patient had not started medication yet, due to his very poor socioeconomic status (which hindered his option of buying the medicines himself) and his nearly nonfunctional health insurance which refused to pay for the drops. He complained of increased eye redness and pain. On clinical examination, there was a 2 × 2 mm descemetocele overlying a large infectious infiltrate with feathery borders that compromised all the way to the sclerocorneal limbus. There was a localized limbal insufficiency. There was an almost complete shallowing of the anterior chamber and a cataract (Figure 1). Due to a complete perforation of the cornea, a standard PACK-CXL was not considered possible, so an emergent, “a chaud,” PK was ordered. Simultaneous noncentered PACK-CXL was also ordered for treatment of the peripheral infiltrate, due to the patient's poor access to medications.

**Figure 2:**
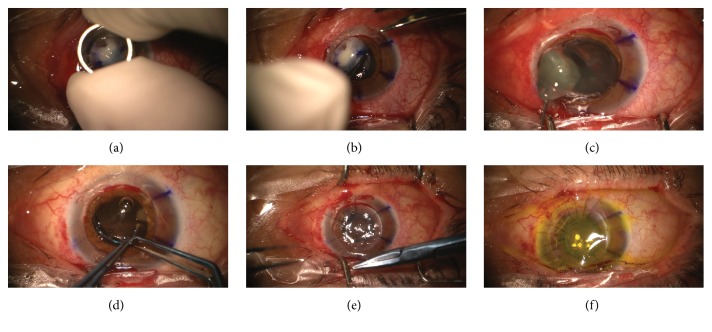
Simultaneous noncentered photoactivated chromophore for keratitis-corneal collagen cross-linking (PACK-CXL) and penetrating keratoplasty. (a) Corneal trephination. (b) Cornea removal. (c) Open-sky cataract removal. (d) Intraocular lens implantation. (e) Donor cornea suturing. (f) Riboflavin impregnation.

**Figure 3:**
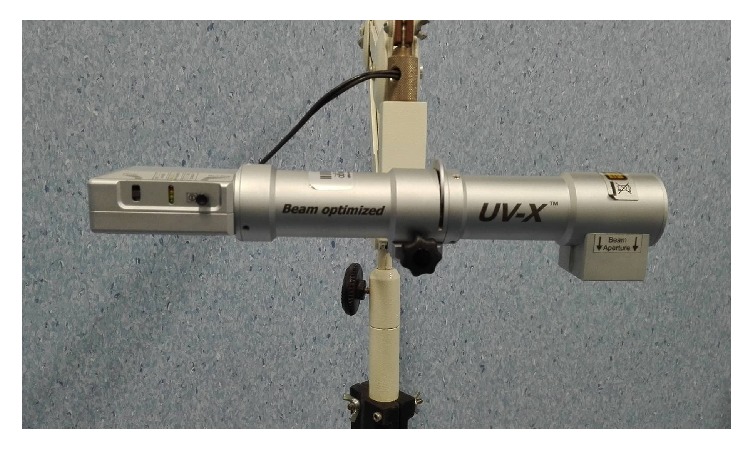
Cross-linking lamp used for the treatment of the patient (UV-X 2000, Avedro Inc., Waltham, United States).

**Figure 4:**
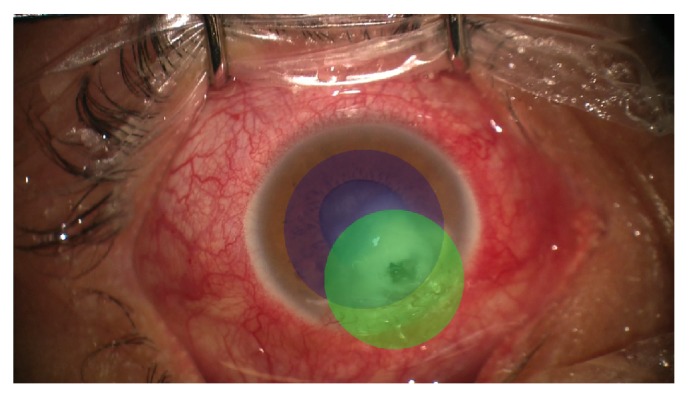
Schematic representation of the areas of the eye covered by the penetrating keratoplasty procedure (blue circle, 8 mm) and the noncentered PACK-CXL (green circle, 7 mm).

Surgery (Figure 2) was performed by one of the authors (K.B.) under general anesthesia: donor and receptor corneas were cut with an 8.50 mm and 8.00 mm trephine, respectively. The cut was centered on the receptor cornea, which was sent for culture. Open-Sky lensectomy was performed with a cystotome, as well as hydrodissection. Aspiration of cortical material was performed with bimanual irrigation/aspiration cannulas, with intraocular lens implantation in the ciliary sulcus, followed by intracameral acetylcholine. Donor cornea was sutured with 10-0 nylon. Then, the eye was impregnated with a solution containing riboflavin 0.1% and dextran 500 (Keralynx®, Nanosigma Biotech Ltd, New Taipei City, Taiwan) at a dose of one application every three minutes for 30 minutes before ultraviolet light exposure. Irradiation with ultraviolet was performed with a CXL machine (UV-X 2000®, Avedro Inc., Waltham, United States) (Figure 3) for ten minutes, aiming for an energy dose of 9 mW/cm^2^. During irradiation, riboflavin irrigation was continued at a rate of one drop every three minutes. An irradiation spot size of 7 mm was used. The patient's eye was grasped superiorly with a 0.30 mm forceps and pulled upwards so the infected area of cornea and limbus was exposed. UV irradiation beam was positioned so that it included part of the donor cornea, the whole infected receptor cornea, affected limbus, and a small part of sclera (Figure 4). After the irradiation, riboflavin was flushed with copious irrigation of balanced salt solution.

**Figure 5:**
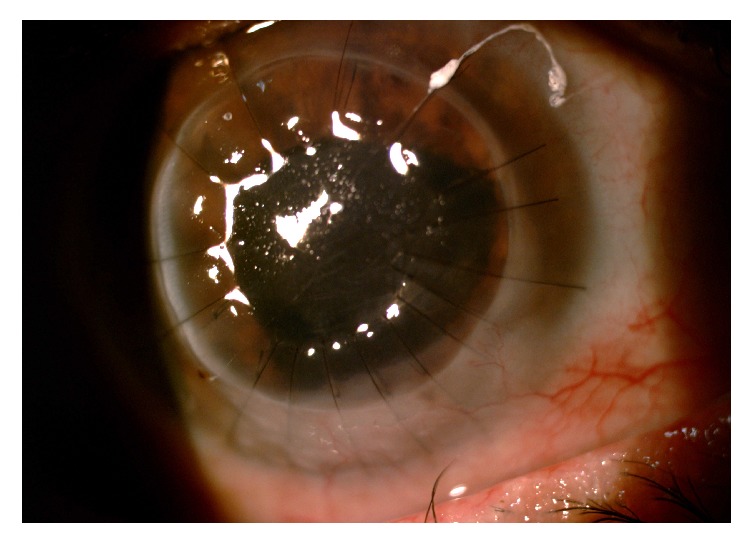
Clinical appearance of the patient three weeks after surgery. Note some natamycin over the eye.

Corneal culture was positive for* Fusarium *spp. Due to the patient's economic status and insurance, he could only start antibiotic and antifungal treatment about 21 days after surgery (Figure 5). His regimen consisted of hourly natamycin, hourly fortified vancomycin, and sodium hyaluronate every two hours. The dose of the drops was slowly tampered.

**Figure 6:**
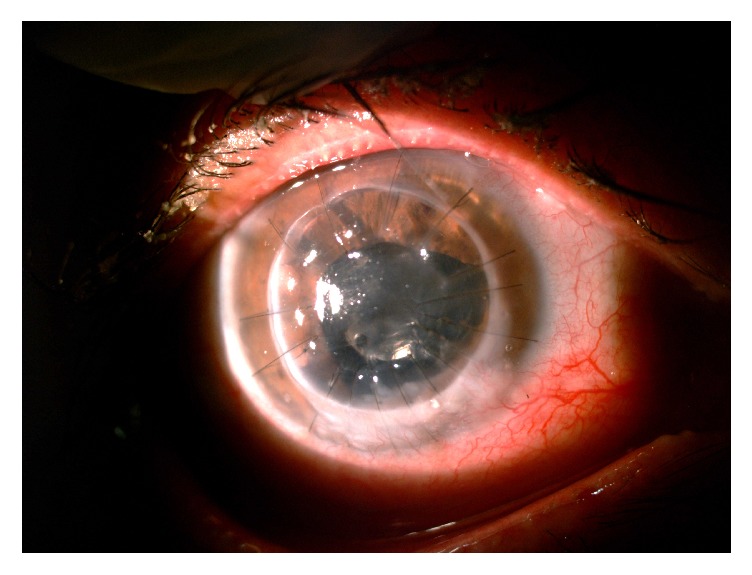
Clinical appearance of the patient two months after surgery, showing a complete resolution of infection, with conjunctivalization replacing the area that was infected in the receptor rim.

During the follow-up period (over four months) the patient has shown an excellent recovery; his eye inflammation has been as expected for a penetrating keratoplasty, and no clinical signs of infection recurrence have been noted (Figure 6). On last clinical evaluation, his pinhole visual acuity was 20/60 (LogMAR 0.47), and he was pain-free, with a clear corneal graft.

## 3. Discussion

The current cross-linking (CXL) technique was first described by Spörl et al. in 1997 [1]; however the antimicrobial properties of riboflavin in combination with UVA light were reported much earlier in 1965 1965 by Tsugita et al. [2]. Since then, it is used for the eradication of microorganisms in water, food, and blood products. Designating the name of photoactivated chromophore for keratitis-corneal collagen cross-linking (PACK-CXL) to the applications of the technique focused on the management of infectious keratitis has been proposed [3, 4]. The mechanism that explains the antimicrobial action is the release of reactive oxygen species and the damage to DNA and RNA of pathogens [5–7]. This effect has been studied in in vitro experiments for both bacterial and fungal keratitis, being higher if riboflavin is combined with UVA light [8]. The cross-linking strengthens corneal collagen degradation by collagenolytic enzymes induced by microorganisms [9]. In addition to these properties, possible nociceptive and anti-inflammatory effects have been reported [10, 11]. Studies in humans have demonstrated the ability of cross-linking to control corneal infections and stop melting in those patients in whom pharmacological management is ineffective. It could also decrease the possibility of a corneal perforation and the need for an emergent penetrating keratoplasty [12–17]. The use of PACK-CXL without antimicrobial agents has also been proposed and its efficacy has been demonstrated in small-sized keratitis [18]. The fungicidal effect has been demonstrated in vitro and in animals for* Candida albicans*,* Fusarium *sp. [19], and* Aspergillus fumigatus* [20], in addition to potentiating the effect of antifungal agents such as amphotericin-B [21]. Studies performed in humans have also demonstrated the effectiveness against fungi [22]. However, there are studies that question its fungicidal effect on deep stromal keratitis [23, 24]. The PACK-CXL protocol has been variable, but most of the studies have used the conventional protocol with irradiation of 365 nm and 3 mW/cm^2^. Some authors have also used an accelerated protocol with irradiation of 365 nm and 9 mW/cm^2^ for 10 minutes [6, 25]. The use of PACK-CXL has been reported in corneas with penetrating keratoplasties with infectious keratitis due to* Staphylococcus aureus*, 12 months after the procedure, with good results [26]. The use of CXL in penetrating keratoplasty has also been reported as a safe method, in addition to hypothetically decreasing the rate of graft rejection [27, 28] and causing a stronger adhesion between donor tissue and receptor [29].

In our case, combining simultaneous noncentered PACK-CXL with centered tectonic penetrating keratoplasty was carried out to treat the fungal marginal keratitis with corneal perforation, in which regular penetrating keratoplasty alone would leave fungal elements in the receptor corneal tissue, which would predispose to infection of the graft. To our knowledge, it is the first reported case worldwide in which the two procedures are done simultaneously, one immediately after the other. PACK-CXL was performed in an decentered way to cover the infiltrates in the lower periphery that could not be removed during the keratoplasty, first performing the keratoplasty to remove the thinned tissue containing the infection, preventing the adverse effects of the UV light on the endothelium [30]. The limbal insufficiency in the inferior zone observed in our case was caused as a consequence of the infection and was present before the surgical procedure. Therefore we can conclude that combining simultaneous noncentered PACK-CXL with centered penetrating keratoplasty appears to be a safe and effective way of treating patients with fungal marginal keratitis with corneal perforation, in which regular penetrating keratoplasty alone would leave fungal elements in the receptor corneal tissue, which would predispose to infection of the graft.

## 4. Conclusions

- Studies in humans have demonstrated the ability of cross-linking to control corneal infections and stop melting in those patients in whom pharmacological management is ineffective. It can also be used to decrease the possibility of requiring an emergent penetrating keratoplasty.
- Combining simultaneous noncentered PACK-CXL with centered penetrating keratoplasty appears to be a safe and effective way of treating patients with fungal marginal keratitis with corneal perforation.
